# Involvement of the 5-HT_1A_ receptor of the cuneiform nucleus in the regulation of cardiovascular responses during normal and hemorrhagic conditions

**DOI:** 10.22038/ijbms.2020.40453.9579

**Published:** 2020-07

**Authors:** Reza Mohebbati, Mahmoud Hosseini, Majid Khazaei, Abolfazl Khajavi Rad, Mohammad Naser Shafei

**Affiliations:** 1 Department of Physiology, Faculty of Medicine, Mashhad University of Medical Sciences, Mashhad, Iran; 2 Division of Neurocognitive Sciences, Psychiatry and Behavioral Sciences Research Center, Mashhad University of Medical Sciences, Mashhad, Iran; 3 Neurogenic Inflammation Research Center, Mashhad University of Medical Sciences, Mashhad, Iran

**Keywords:** Hemorrhage, Mean arterial pressure, Medullary nucleus, Serotonin receptor, Systolic blood pressure

## Abstract

**Objective(s):**

The 5-hydroxytryptamine1A (5-HT_1A_) receptor is one of the serotonin receptors in the brain, which regulates cardiovascular responses, especially in hemorrhage. Presence of this receptor in the cuneiform nucleus (CnF) has been shown. The present study evaluates the cardiovascular effect of this receptor of the CnF in normal and hypotensive hemorrhagic rats.

**Materials and Methods:**

Agonist (8-OH-DPAT) and antagonist (WAY-100635) of 5-HT_1A_ microinjected into the CnF in basal and hemorrhagic conditions and cardiovascular responses were evaluated. Hemorrhage induced by blood withdrawal from the femoral artery and 2 min after that drugs microinjected. Time course and peak changes (∆) of the mean arterial pressure (MAP), systolic blood pressure (SBP) and heart rate (∆HR) were obtained and compared to the control and hemorrhage groups.

**Results:**

In basal condition, 8-OH-DPAT significantly decreased ∆SBP, ∆MAP and ∆HR compared to the control (*P<*0.05-*P<*0.01), while way-100635 did not have a significant effect. Hypotension and tachycardia induced by hemorrhage ameliorated by agonist (*P<*0.05-*P<*0.01), while antagonist deteriorated hypotension (*P<*0.05) but attenuated tachycardia (*P<*0.01).

**Conclusion:**

This study shows that 5-HT_1A_ receptor of the CnF involves in regulation of the cardiovascular responses. However, this effect in basal and hemorrhage conditions is different.

## Introduction

Serotonin (5-hydroxytryptamine, 5-HT) is a monoamine neurotransmitter that is synthesized in the peripheral and central nervous system (CNS) (1, 2). In the CNS, the 5-HT plays an important role in functions such as mood, appetite, sleep, and memory, and its reduction causes several problems, especially depression (3). Central cardiovascular effect of the serotonergic system has also been reported previously (2). 

Serotonergic neurons by projecting to important cardiovascular areas such as rostral ventrolateral medulla (RVLM), nucleus tractus solitari (NTS) and paraventricular nucleus of hypothalamus (PVN) or direct projecting to intermediolateral cell column (IML) (1, 2, 4) precipitate in cardiovascular regulation. Due to various receptors of 5-HT, its effect on the cardiovascular system is complicated and could evoke bradycardia/tachycardia or hypotension/hypertension responses (1, 5). One of the important receptors of 5-HT is 5-HT_1A_ that its effects on cardiovascular responses in basal and hemorrhagic (HEM) conditions has been reported (1, 6). In normotensive state microinjection of 8-hydroxy-2-(di-n-propylamino)-tetralin (8-OH-DPAT), a selective agonist of 5-HT_1A_–into the RVLM and ventrolateral periaqueductal gray matter (vlPAG) inhibits cardiovascular responses (7, 8). In HEM, it can reverse hypotension induced by the HEM (1). 5-HT_1A_ receptor is also in other nuclei such as the cuneiform nucleus (CnF) (9). 

The CnF is a mesencephalic nucleus located on the anterolateral of the PAG and precipitates in various functions including modulation of pain, regulation of movement, respiration, stress, and sleep (11-14) and cardiovascular regulation (15). Lam *et al.* reported that electrical stimulation of the CnF elicited sympathetic vasomotor outflow and pressor effect (16). Moreover, Korte *et al.* proposed that the CnF was located in the center of a circuit that by affecting sympathetic or parasympathetic systems regulates cardiovascular responses to stress. The presence of several neurotransmitters such as acetylcholine (Ach), glutamate, gamma aminobutyric acid (GABA), nitric oxide (NO) and serotonin (5-HT) (9, 13) has been reported in the CnF nucleus. From them, the cardiovascular effect of glutamatergic, cholinergic and nitrergic systems has been evaluated in our previous studies (17-19). There is also evidence that the CnF involved in cardiovascular regulation during the HEM condition. 

It has been shown that HEM significantly increased Fos-like immunoreactivity (FLI) in the CnF (20). In addition, relation of the CnF with numerous brain areas involved in regulation of the HEM such as RVLM (10), NTS (21) and hypothalamus (10, 22, 23) areas has also been indicated. In a preliminary study, we showed the involvement of this nucleus in the HEM after blocking the nucleus with cobalt chloride (CoCl_2_, a synaptic blocker). Based on this evidence, we suggest that the CnF involves in cardiovascular regulation during the HEM condition. Because of the 5-HT_1A_ receptor present in the CnF (24), this study designed to determine the cardiovascular effect of this receptor in normal and hemorrhagic conditions in anesthetized rats.

## Materials and Methods

***Animals***

Thirty six male Wistar rats (250±20 g) were used in this experiment. The rats were maintained in a room at 21±3 ^°^C under a light-dark cycle of 12:12 hr with food and water available *ad libitum*. The temperature of animals was maintained at 37 ^°^C with a heating lamp. All procedures were performed in accordance with the Bioethics Committee of the Mashhad University of Medical Sciences guidelines for the care and use of experimental animals (IR.MUMS.fm.REC.1395.585).

***Surgery and microinjection of drugs***

To record cardiovascular responses, the animals were anesthetized with urethane (1.5 g/kg, IP). Then, the left femoral artery was cannulated with a 22-gauge Angiocath catheter (Indian Co) filled with heparinized saline. It was connected to a blood pressure transducer, and systolic blood pressure (SBP), mean arterial pressure (MAP) and heart rate (HR) were continuously recorded by a power lab system (ID instrument, Australia) (25). The right femoral artery was also cannulated for blood withdrawal.

For microinjection, the animals were placed in a stereotaxic apparatus (Stoelting, USA). The scalp was incised and the skull was leveled between lambda and bregma, and a small hole was drilled in the skull above the CnF according to the Paxinos and Watson atlas. The stereotaxic coordinates of the CnF were -7.6 to -8.5 mm caudal to the bregma, -1.7 to -2.2 mm lateral to the midline suture and -5.5 to -6.2 mm ventral from the bregma (26). Microinjection of agonist/antagonist of the 5-HT_1A_ receptor into the CnF was performed by a single barreled micropipette with 40 μm diameter. The micropipette was connected to a manual microinjector (Harvard) through a PE-10 tube and carefully introduced into the CnF; the injection was performed during 30 sec. The injection volume in all groups was 100-150 nanoliter (25). 

***Hemorrhage protocol***

 For induction of hemorrhage, an arterial catheter filled with heparinized saline (50 ul/ml) was used to prevent clotting during blood withdrawal. In this experiment, about 15% of total blood volume (TBV) was withdrawn (4). The amount of 15% reduction of blood is an intermediate hemorrhage that could reduce systolic blood pressure about 30-35 mmHg, a suitable condition to evaluate cardiovascular responses in brain areas during the HEM condition (20). The following formula was used for calculation of the TBV (ml): 0.06 (ml/g)×body weight (g)+0.77 (20). 

After mounting the animals on the stereotaxic apparatus and stabilizing the hemodynamic parameters, blood was taken from the rat artery with the rate of 1 ml/100 g body weight over 10 min (7). Two minutes after induction of the HEM condition (12 min after initiating hemorrhage), drugs were microinjected into the CnF and changes of cardiovascular responses were determined. 

***Drugs and animal groups***

The drugs included urethane, 8-hydroxy-2-(di-n-propylamino)-tetralin (8-OH-DPAT), a selective 5-HT_1A_ agonist and WAY-100635, a 5-HT_1A_ antagonist provided from Sigma, USA. The solvent of all drugs was saline.

The experimental groups were as follows (n=6 in each group):

1) Control: Microinjection of saline into the CnF

2) 8-OH-DPAT group: Microinjection of 8-OH-DPAT (OH, 10 nmol) into the CnF

3) WAY-100635 group: Microinjection of WAY-100635 (WAY, 3 nmol) into the CnF

4) Hemorrhage (HEM): Microinjection of saline into the CnF 2 min after HEM (blood withdrawal (1 ml/kg/BWT) over 10 min) 

5) 8-OH-DPAT+HEM: Microinjection of OH-DPAT into the CnF 2 min after the HEM

6) WAY-100635+HEM: Microinjection of WAY into the CnF 2 min after the HEM

The volume of injection in all groups was 100-150 nl.

***Data analysis***

The data were calculated and expressed as mean±SEM. For evaluation trend of responses, changes (∆) of SBP, MAP, and HR were obtained after injection of drugs in several times and compared to changes in the control group (repeated measures ANOVA). In the HEM groups, changes in all the parameters after injections of drugs were also compared to those in the HEM groups. In addition, to find maximal responses of drugs, peak changes of ∆SBP, ∆MAP, and ∆HR of each group were also separately provided about 20 min after termination of the HEM and drug injection. All peak changes were compared to control or HEM groups (One-way ANOVA, tukey’s *Post hoc* test). A value of *P*<0.05 was used to indicate statistical significance.

***Histology***

**Figure 1:**
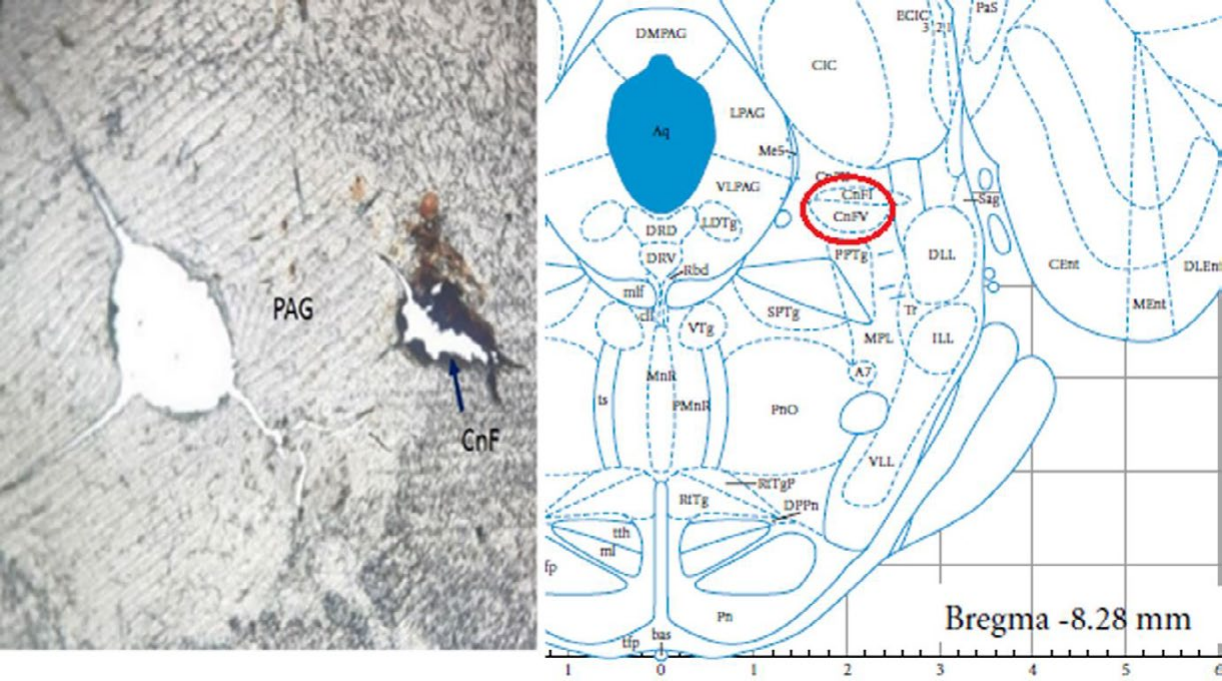
A photograph of the brain at the CnF level that show injection site of drug

PAG: periaqueductal gray matter; CnF: Cuneiform nucleus

At the end of each experiment, the rats’ brains were removed and after being fixed by formalin 10%, serial slices with 50-micron thickness were prepared using a vibrating microtome (ESM Co, USA). Verification of injection sites was performed by direct observation of slices under a light microscope (27). A sample of an injection site is indicated in Figure 1.

## Results

***Cardiovascular responses to microinjection saline into the CnF nucleus in normotensive rats***

In normal condition, saline was microinjected into the CnF and cardiovascular parameters were evaluated before and after microinjection. The basic values of the MAP, SBP and HR were 112.7±0.56 mmHg, 118±1.32 mmHg, and 341.6±5.31 beats/min, respectively that those changes were not significant compared to basic values (∆MAP: -3.1±1.9 mmHg, ∆SBP: -4.2±1.5 mmHg and ∆HR: -5.5±2.8 beats/min).

***Cardiovascular responses to microinjection of 8-OH-DPAT and WAY-100635 into the CnF in normotensive rats***

**Figure 2:**
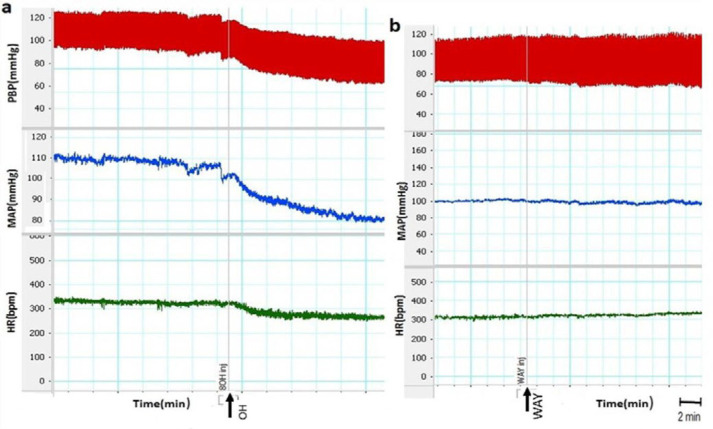
The samples of PBP, MAP and HR recorded after microinjection of the OH and WAY into the cuneiform nucleus in normotensive rats. Time of injection is marked by the vertical line

**Figure 3:**
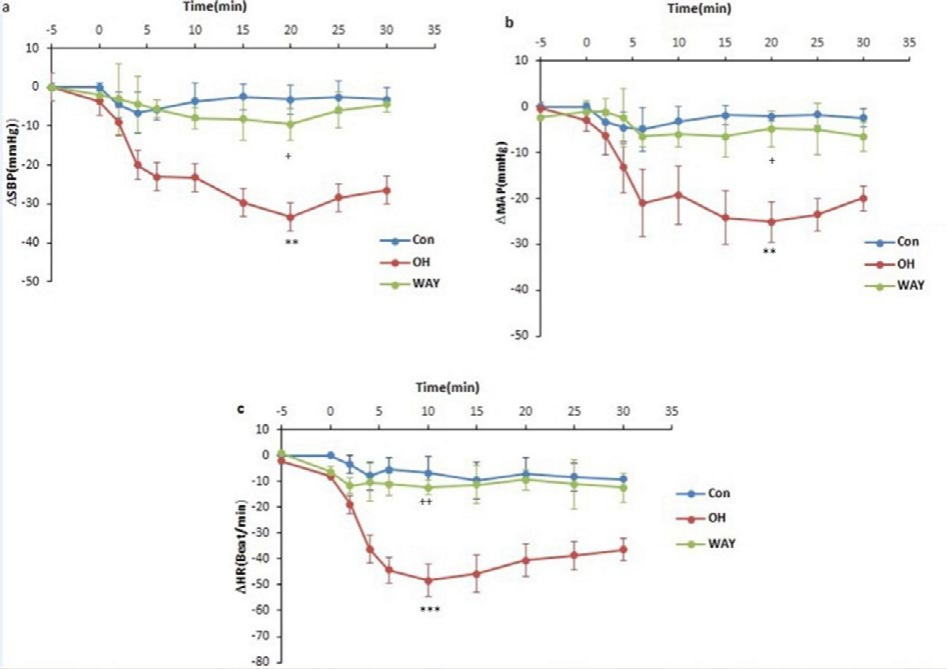
Time course and peak changes of ∆SBP (a), ∆MAP (b) and ∆HR (c), after microinjection of the agonist (OH) and antagonist (WAY) of 5-HT_1A_ receptor into the cuneiform nucleus. In OH group, ∆SBP, ∆MAP and ∆HR significantly decreased compared to the control over time (repeated measures ANOVA, *P<*0.01 to *P<*0.001)

In this study, to examine the role of 5-HT_1A_ receptor of the CnF on cardiovascular responses, the OH and WAY were microinjected into the CnF. Our results indicated that the OH decreased all the cardiovascular parameters (Figure 2). Time-course changes in cardiovascular responses in the OH group are shown in Figure 3. ∆MAP, ∆SBP, and ∆HR in the OH group significantly declined compared to the saline group over time (repeated measures ANOVA, *P*<0.01 to *P*<0.001). However, microinjection of WAY did not significantly affect the parameters compared to saline over time (repeated measures ANOVA, *P*>0.05, Figure 3). The peak ∆SBP, ∆MAP, and ∆HR were also calculated and compared to changes in saline. In the OH group, ΔSBP (OH: -33.4±6.4 mmHg vs saline: -4.2±1.5 mmHg, *P*<0. 01), ΔMAP (OH: -25.1±5.9 mmHg vs saline: -3.1±1.9 mmHg, *P*<0. 01) and ΔHR (OH: -48.4±8.4 beats/min vs saline: -5.5±2.8 beats/min; *P*<0.001; One-way ANOVA) significantly decreased compared to those in the saline group (Figures 3). In the WAY group, peak cardiovascular changes were not significant compared to the control group ∆SBP (WAY: -9.5±5.8 mmHg vs saline: -4.2±1.5 mmHg), ΔMAP (WAY: -4.75±4.4 mmHg vs saline: -3±1.9 mmHg) and ΔHR (WAY: -11.2±4.3 beats/min vs saline: -5.5±2.8 beats/min; One-way ANOVA; Figure 3). All parameters in OH group also were significant respect to WAY group (*P<*0.05 to *P<*0.01).

***Cardiovascular responses during induction of hypotensive hemorrhage***

**Figure 4:**
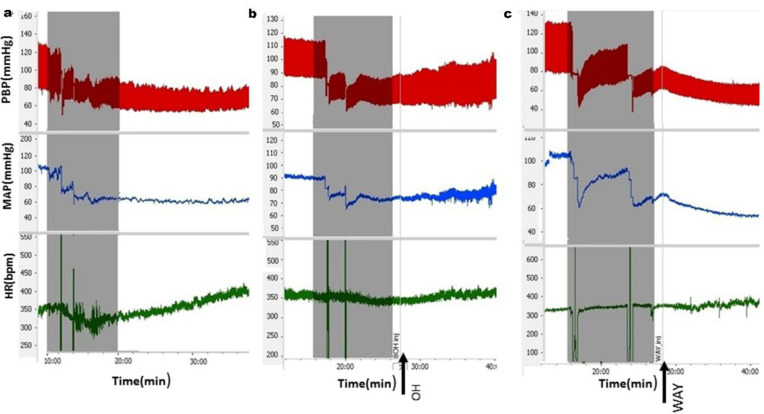
The samples of PBP, ∆MAP and HR recorded after hemorrhage (a) microinjection of the OH (b) and WAY(c) into the cuneiform nucleus in hypotensive hemorrhagic rats. Time of injection is indicated by an arrow

**Figure 5:**
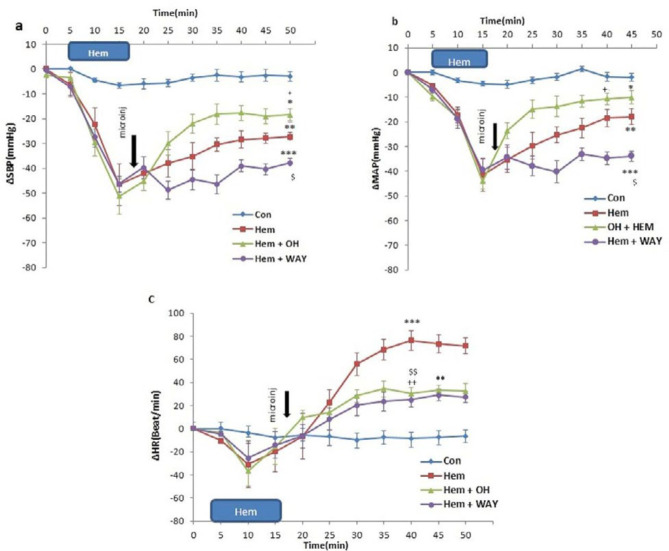
Time course and peak changes of ∆SBP (a), ∆MAP (b) and ∆HR (c), after microinjection of the agonist (OH) and antagonist (WAY) of 5-HT_1A_ into the cuneiform nucleus in hypotensive hemorrhagic rats. Microinjection of the OH significantly attenuated hypotension and tachycardia induced by hemorrhage over time (repeated measures ANOVA, *P<*0.01 to *P<*0.001)

To induce the HEM, about 15% of the TBW of blood was taken over 10 min. The HEM caused decrease in the SBP, MAP, and HR. After that, the SBP and MAP were slowly returned and stabilized lower than a basal state within 20 min. HR also gradually increased and was stabilized higher than the basic state (Figure 4A). Time course of changes after the HEM showed a significant reduction in the MAP and SBP compared to the control group over time (repeated measures ANOVA, *P*<0.01, n= 6, Figure 5). Moreover, the HR was significantly lower compared to the control group over time (repeated measures ANOVA *P*<0. 001, n=6). 

Peak changes of cardiovascular parameters were calculated after termination of the HEM and compared with changes of the control group. Results revealed that ΔSBP (HEM: -31.4±4.6 mmHg vs. saline: - 3.6±1.8 mmHg, *P*<0.01) and ΔMAP (HEM: -26.6±4 mmHg *vs* saline: -2.1±1.9 mmHg, *P*<0.01) significantly decreased whereas ΔHR significantly increased (HEM: 65.2±8.7 beats/min *vs* saline: -8.4±5.6 beats/min; *P*<0.001; One-way ANOVA, Figure 5) compared to the saline group. 

***Cardiovascular responses to microinjection of 8-OH-DPAT and WAY-100635 into the CnF after induction of hypotensive hemorrhage***

In this experiment, to determine whether the 5-HT_1A_ receptor agonist and antagonist affect cardiovascular responses induced by the HEM, agonist (OH) and antagonist (WAY) of receptor were microinjected 2 min after HEM into the CnF (Figure 4B and C). Time-course changes of the SBP, MAP, and HR in the HEM+OH and HEM+WAY groups are shown in Figure 5. As indicated, in the HEM+OH group, the OH could ameliorate hypotension induced by HEM over time (repeated measures ANOVA, *P<*0.05, Figure 5) and the HR significantly decreased compared to the HEM group (repeated measures ANOVA, *P*<0.01, Figure 5). In the WAY+HEM group, WAY deteriorated hypotension (*P<*0.05) and reduced HR over time (*P<*0.01). The peak changes after microinjection of the OH and WAY indicated that in the HEM+OH group, only ∆SBP (OH+HEM: -10.68±4.3 mmHg vs. HEM: -26.6±4.1 mmHg, *P* <0.05 and HR: OH+HEM: 30.4±5.30 beats/min vs. HEM: 65.2±8.7 beats/min, *P*<0.01, one-way ANOVA) was significant compared to the HEM group (Figure 5).

In the WAY+HEM group, peak changes of ∆SBP (WAY+HEM: -27.2±2.8 mmHg vs. HEM: -2.3±1.9 mmHg, *P*<0.05) and ∆MAP (WAY+HEM: -27.2±2.8 mmHg vs. HEM: -2.3±1.9 mmHg, *P*<0.05) were significantly lower in comparison with the HEM group. Moreover, peak ∆HR was significantly reduced compared to the HEM group (WAY+HEM: 24.93±6.2 beats/min vs. HEM: 65.2±8.7 beats/min, *P*<0. 01, One-way ANOVA; Figure 5).

## Discussion

The results revealed that in normotensive rats, cardiovascular parameters significantly attenuate by microinjection of 8-OH-DPAT into the CnF. However, in the HEM condition, the OH reversed hypotension and tachycardia induced by the HEM, whereas the WAY deteriorated hypotension but attenuated tachycardia.

The central cardiovascular effect of 5-TH_1A_ receptor was evaluated in several brain areas such as RVLM and vlPAG. These experiments showed that OH could significantly decrease cardiovascular parameters (8, 28, 29). In the line with these studies, our results indicated the significant decrease of all cardiovascular parameters.

Mechanism of the cardiovascular inhibitory effect of this receptor of the CnF is unknown, but several mechanisms are possible. The CnF is a heterogeneous nucleus which, in addition to having the cardiovascular effect, is involved in several functions such as movement, sleep and pain modulation (11, 13, 14). In cardiovascular function, it has been proposed that the CnF is placed in the center of a circuit that has two sympathetic and parasympathetic limbs and mediates cardiovascular responses to threaten stimuli and stressors (15). Therefore, the cardiovascular effect of the OH may be mediated by each one of the sympathetic or parasympathetic systems.

In the sympathetic context, the CnF has a sympathoexcitatory effect by activating neurons of the RVLM (15, 30). Since the 5-HT_1A_ receptor is an inhibitory receptor that is coupled to G_i_ of the G-protein complex and reduces cardiovascular parameters (2, 7), we suggest that injection of the OH into the CnF by hyperpolarization of neurons, causes to decline the sympathetic excitatory drive from the CnF to the RVLM and reduce cardiovascular parameters. 

Although the cardiovascular effect of the CnF is mostly mediated by the RVLM, its direct projection to the RVLM is spare and its relation is mostly polysynaptic and mediated by other areas such as kolliker fuse (KF), dorsolateral PAG (dlPAG) and raphe nuclei (12, 15, 30). Chemical and electrophysiological studies showed that activation of the CnF by glutamate produced two different short (pressor and bradycardia) and long (pressor and tachycardia) responses, and short responses blocked by inactivation of the KF, while long responses did not change (17). 

As in the present study, microinjection of the OH into the CnF decreased blood pressure and HR for a long time; it is conceivable that the inhibitory effect of the OH was mostly mediated via the long response. Since the long response was not affected by the KF nucleus, we suggest that this response was mediated by other nuclei such as raphe nuclei or dlPAG (10, 23). Raphe nuclei are involved in cardiovascular regulation and these effects are partly mediated by projection to the RVLM. Therefore, we suggest that cardiovascular effect of 5-HT_1A_ receptor in the CnF is mediated by CnF-Raphe- RVLM pathway (12). 

In this study, blockade of the 5-HT_1A_ receptor by the WAY, did not have a statistically significant effect on the cardiovascular parameters. As previously reported that glutamatergic receptors of the CnF did not involve in basal cardiovascular condition, we suggest that in the anesthesia condition, the release of 5-HT is also probably very low. Therefore, antagonist did not affect the cardiovascular system.

In the parasympathetic context, the CnF sends descending fibers to important parasympathetic brain areas such as gigantocellular reticular nuclei, motor nucleus of the vagus (DMNV) and NTS (15). Since pressor and HR in the CnF are separately regulated, it is possible that projections to these areas are involved in the bradycardia induced by OH. However, future studies must be carried out to further explore this issue. 

The CnF has several neurotransmitters such as glutamate, GABA, and NO (9); therefore, the interaction of 5-HT_1A_ with these neurotransmitters is also probable. It has also been reported that 5-HT_1A_ was expressed pre-synaptically on non-serotonergic neurons and inhibited the release of neurotransmitters (2). Due to the excitatory effect of glutamate in the CnF, we proposed that inhibitory effect of 5-HT_1A_ was mediated by reduction in the release of glutamate. 

In another experiment, we evaluated the cardiovascular effect of 5-HT_1A_ receptor in the HEM condition. Microinjection of OH into the CnF could reverse the hypotensive effect of the HEM, while the WAY deteriorated this effect. 

The HEM is a complicated condition, involving several neurotransmitters, especially serotonin. The 5-HT_1A _receptor is one of the well-known receptors of serotonin that could improve cardiovascular responses in the HEM. Our present findings are also consistent with the aforementioned studies, showing that activation of 5-HT_1A_ of the CnF reverses the hypotensive effect of the HEM. The mechanism of the effect of 5-HT_1A_ in the CnF has not been yet clarified. However, several mechanisms may be involved. The initial response in HEM is activation of baroreflex to maintain blood pressure in basal condition. Since the anatomical relation of the CnF with brain areas involved in baroreflex (i.e., the NTS and CVLM) has been reported, it is conceivable that during the HEM, the 5-HT_1A_ receptor by suppressing inhibitory effects of the CVLM on RVLM neurons, could recover hypotension and tachycardia evoked by the HEM. Therefore, the effect of the 5-HT_1A_ receptor is different in normotensive and HEM conditions. 

Chemoreflex responses also contribute to control of cardiovascular function in the HEM condition. Therefore, it is possible that the 5-HT_1A_ receptor of the CnF precipitates in the regulation of cardiovascular responses by affecting nuclei or pathways involved in chemoreflex regulation. However, future studies are needed to clarify these issues. 

In addition to the effect of reflexes, as previously mentioned, a glutamatergic projection from the CnF to raphe nuclei has also been reported (32). Lam *et al. *also indicated that pressor induced by the electrical activation of the CnF was attenuated by antagonist injection of the 5-HT antagonist into the spinal cord. As raphe nuclei are the main area for synthesis of 5-HT, we suggest that this pathway also precipitates in the regulation of cardiovascular regulation during the HEM. Therefore, CnF-raphe-RVLM–spinal or CnF-raphe- spinal pathway may mediate the effect of CnF during the HEM. 

A relation of the CnF to the PVN has also been reported previously (23). The 5-HT_1A_ receptor has also been shown in PVN. Because PVN neurons beside regulation of cardiovascular responses are also involved in body fluid homeostasis, by release of circulating peptides (vasopressin), we suggested that the CnF projections to the PVN improve the cardiovascular response to the HEM. However, further studies are needed to clarify the issue.

In addition, we previously indicated that the cholinergic and nitrergic system of the CnF significantly decreased blood pressure with no effect on the HR (18, 19). Therefore, it is possible that activation of 5-HT_1A_ receptors during the HEM pre-synaptically decrease the release of these neurotransmitters (33). Therefore, the inhibitory cardiovascular effect of cholinergic or nitrergic systems decreased by disinhibition phenomena and evoked cardiovascular responses. The GABAergic neuron has also been shown in the CnF (13), and it is possible that 5-HT_1A_ decreased the inhibitory effect of the GABAergic system and accelerated the recovery of cardiovascular responses during the HEM.

The CnF was also reported to be involved in the integration of threatening and stressful stimuli (15). Accordingly, the CnF might integrate in the cardiovascular response to pain, fear, exercise, and other stressors. Since the HEM is also a threatening factor, we suggest in this condition that the CnF receives projections from the cortex, PVN and dorsomedial hypothalamus and modulates cardiovascular parameters. One of the receptors involved in this effect is probably the 5-HT_1A_ receptor. However, future studies are needed to confirm this effect.

In this study, microinjection of the WAY into the CnF deteriorated the hypotensive effect during the HEM, confirming the recovery effect of the 5-HT_1A_ receptor. However, the WAY decreased tachycardia induced by the HEM. This effect may be mediated by activation of the parasympathetic system via projection of the CnF to the NTS or vagal nuclei. 

Our results also revealed that both the agonist and antagonist of 5-HT_1A_ could attenuate tachycardia induced by the HEM. However, the mechanism of this effect was not determined, which needs to be evaluated in future studies. 

PBP: Pulsatile blood pressure, MAP: Mean arterial pressure, HR: Heart rate, OH: 8-OH-DPAT, WAY: WAY-100635

**P<0.05 and ***P<0.001 peak changes vs control group (One-way ANOVA, tukey’s *Post hoc *test)

++*P<*0.01 peak changes of OH compared to WAY (One-way ANOVA, tukey’s *Post hoc* test)

The data were expressed as mean±SEM; n= 6

∆SBP: Systolic blood pressure, ∆MAP: Mean arterial pressure, ∆HR: Heart rate, Con: control, OH: 8-OH-DPAT, WAY: WAY-100635

PBP: Pulsatile blood pressure, ∆MAP: Mean arterial pressure, HR: Heart rate, OH: 8-OH-DPAT, WAY: WAY-100635

The data were expressed as mean±SEM; n=6

*P<0.05,**P<0.01 and *P<*0.001 peak changes vs control group (One-way ANOVA, tukey’s *Post hoc* test)

+*P<*0.05 and ++*P<*0.01 peak changes of HEM+OH compared to HEM (One-way ANOVA, tukey’s *Post hoc* test) ^$^*P<*0.05 and ^$$^ *P<*0.01 peak changes of Hem+WAY compared to HEM

∆SBP: Systolic blood pressure, ∆MAP: Mean arterial pressure, ∆HR: Heart rate, Con: control, OH: 8-OH-DPAT, WAY: WAY-100635, Hem: Hemorrhage

## Conclusion

Our results in first time indicated that the 5-HT_1A_ receptor of the CnF was involved in regulation of the cardiovascular system both in normal and HEM conditions but its effects in two conditions are different.
